# Pyridine in Asthma

**Published:** 1887-06

**Authors:** 


**Pyridine in Asthma.**—The *Quarterly Therapeutic Review* contains the following extract from the *Union Medicale:* "Pyridine is valuable as an anti-asthmatic, whether the affection is of cardiac origin, or otherwise. About a drachm of the drug is placed on a plate in a small room, to which the patient pays periodical visits of from twenty to thirty minutes' duration three times a day. After two or three *seances* the rales in the chest disappear, the expectoration is more free, and sleep is obtained at night, or, at all events, relief from the asthmatic attacks. In some cases the improvement is permanent, in others it only lasts unimpaired for five or six days. Iodine treatment is then required, which is usually efficacious, but which can not be borne by all patients."—*National Druggist.*

